# Carcinosarcoma of the fallopian tube: A rare case with rapid metastases and port-site metastasis

**DOI:** 10.1016/j.ijscr.2025.110889

**Published:** 2025-01-13

**Authors:** Takuya Kuboya, Kaoru Kato, Junichi Sasaki

**Affiliations:** Department of Obstetrics and Gynecology, Moriya Daiichi General Hospital, Moriya, Ibaraki, Japan

**Keywords:** Fallopian tube cancer, Carcinosarcoma of the fallopian tube, Port-site metastasis, PSM excision, Case report

## Abstract

**Introduction and importance:**

Fallopian tube cancer, particularly the carcinosarcoma subtype, is a rare malignancy posing diagnostic challenges.

**Case presentation:**

Our patient was an 83-year-old, nulligravida woman, presented to our outpatient clinic with one month of pelvic pain. On examination, a pelvic mass was detected. MRI suggested a cystic tumor in the fallopian tube, and pyosalpinx was initially suspected. Laparoscopic surgery was performed. However, she was later diagnosed with carcinosarcoma of the fallopian tube, according to the pathological diagnosis. The disease rapidly recurred and metastasized, including the development of Port-Site Metastasis (PSM). The patient underwent PSM excision surgery based on her request, which contributed to her mental well-being.

**Clinical discussion:**

Diagnosing fallopian tube cancer is difficult, and there is often a misdiagnosis risk during surgery, leading to potential errors in treatment. MRI can help identify fallopian tubal cancer, but its features can also resemble benign conditions. Factors like infertility and chronic inflammation can increase the suspicion of cancer. Preventive measures for PSM during laparoscopic surgery are crucial. While it's unclear if removing port-site metastases improves the prognosis, it may benefit the patient's mental health.

**Conclusion:**

Our findings underscore the importance of considering the possibility of malignancy when treating tubal tumors. We should take preventive measures for PSM during laparoscopic surgery. PSM excision surgery could be an option to enhance patient's mental health.

## Highlights

• Fallopian tube cancer, especially carcinosarcoma subtype, is exceptionally rare and challenging to diagnose and treat.
• When managing tubal tumors, it’s essential to maintain a suspicion of malignancy and perform at least a biopsy during surgery.
• Surgeons should also consider the potential for PSM and take measures to prevent it.
• PSM excision surgery may provide significant psychological support.

## 1 Introduction

Fallopian tube cancer represents a rare malignancy, within its subtype, carcinosarcoma is even more uncommon [1]. It is a challenging disease to diagnose and manage due to its difficulty in distinguishing from benign tumors and its tendency for high malignancy and rapid progression. We report a case of fallopian tube carcinosarcoma diagnosed after laparoscopic surgery, which subsequently followed a rapid course of metastasis and recurrence. The work has been reported in line with the SCARE criteria [2].

## 2 Case presentation

Patient aged 83 years old, nulligravida, having a history of surgery for appendicitis. In her 30s, she was diagnosed with bilateral fallopian tube blockage due to chlamydia infection.

She initially sought medical attention for pelvic pain, but the decision was made to observe without intervention, resulting in no improvement of pain. One month later, she independently sought out and visited our hospital for her initial consultation. At the time of examination, she complained of intermittent lower abdominal pain. Physical examination and transvaginal ultrasound test revealed a pelvic mass, measuring 10 cm, which was painful to the touch. Blood tests showed only a mild elevation of white blood cell (WBC) to 9400 /μL (normal <8600 /μL) and c-reactive protein (CRP) to 4.23 mg/dL (normal <0.5 mg/dL). Other blood test results were unremarkable. Among tumor markers, only cancer antigen 19–9 (CA19–9) was slightly elevated at 90.0 U/mL (normal <37 U/mL).

**Fig. 1:**
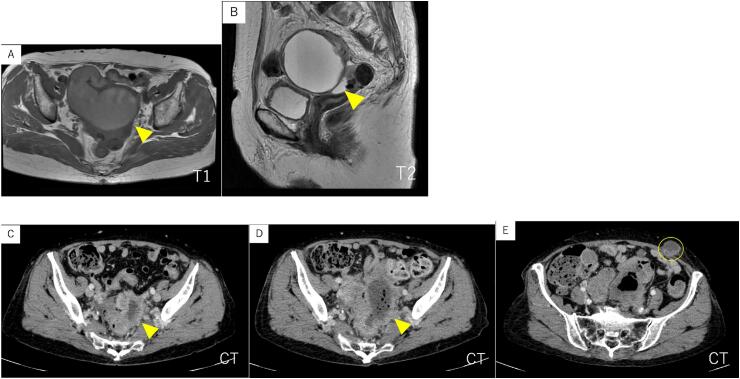
Images of the patient. A: MRI, T1-weighted image. Sausage-shaped fallopian tube present. B: MRI, T2-weighted image. Cystic lesion present ventral and cranial to the uterus. C: CT, fluid accumulation present on the left side of the sigmoid colon. D: CT, increased fluid accumulation on the left side of the sigmoid colon, with gas present internally. E: CT, mass formation presence at the left lower abdominal port site.

A pelvic MRI was performed showing a cystic tumor with a maximum diameter of approximately 10 cm, having tortuous tubular structures and suspected to involve the fallopian tube. There is no clear enhancement of the solid components or wall thickening in either T1 or T2 imaging (Fig. 1A, B). These findings raised the suspicion of pyosalpinx resulting from a fallopian tube infection.

**Fig. 2:**
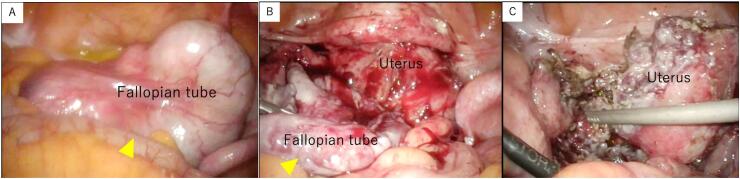
Laparoscopic images. A: Enlarged left fallopian tube. B: Separation of adhesions between the uterus and the fallopian tube. C: After resection.

About two months after the first visit, Laparoscopic surgery was performed for pyosalpinx. It showed the left fallopian tube was enlarged and firmly adhered to the sigmoid colon, rectum, and uterus, being embedded, the right adnexa was normal (Fig. 2A). Bilateral adnexectomy was performed. During the procedure, there was a leakage of the contents of the left fallopian tube, which appeared as brown purulent fluid. Due to strong adhesions, a partial residual portion was left in place, and tumor resection was performed to the possible extent (Fig. 2C).

The postoperative course was straightforward. The patient was discharged on the 6th day of post-surgery.

**Fig. 3:**
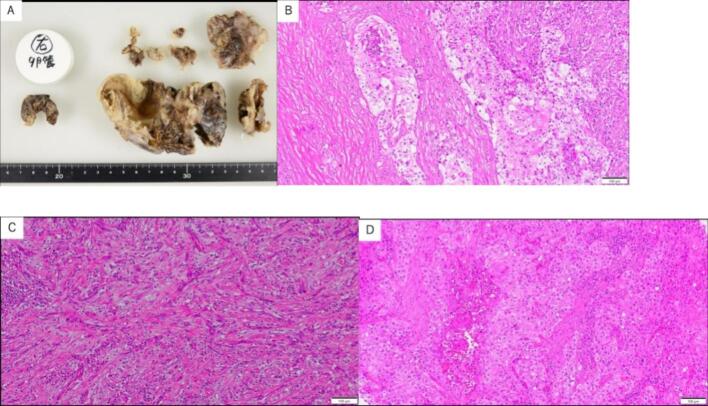
Specimen and pathological images. A: Gross image. Thickening of the fallopian tube wall is observed. B: Initial surgical specimen, undifferentiated high-grade epithelial component, Hematoxylin-eosin(HE) stain, 100×. C: Initial surgical specimen, fibrosarcomatous stromal component, HE stain, 100×. D: Recurrent tumor specimen, cells similar to the primary tumor HE stain, 100×.

One month after the surgery, pathological diagnosis revealed it to be Carcinosarcoma, with pyosalpinx, left fallopian tube (Fig. 3A). It consisted of an epithelial component composed of highly malignant cells of unclassifiable type (Fig. 3B) and a stromal component resembling fibrosarcoma (Fig. 3C). The right adnexa was free of tumor. Modified International Federation of Gynecologic Oncology (FIGO) stage was IC1 or more because of inadequate surgery. Taking into consideration that the patient was over 80 years old, living alone without any relatives to support her treatment, and the fact that it is a refractory condition, the decision was made not to pursue aggressive treatment, even in case of recurrence, but rather to opt for palliative care when symptoms arise.

Two months after the surgery, CT showed recurrence tumor, corresponding to the tumor excision site on the left pelvic wall (Fig. 1C). Diarrhea and lower abdominal pain gradually intensified. For inadequate pain control with NSAIDs, the patient began using narcotic medications.

Subsequent CT scans due to acute abdominal pain a few months later showed that having known left pelvic mass making a fistula with the sigmoid colon (Fig. 1D) and abdominal mass located directly beneath the left upper abdomen laparoscopy port site. The overall condition improved with antibiotic administration.

However, there was a gradual increase in Port-Site Metastasis (PSM) (Fig. 1E). With the patient's consent and desire, tumor resection surgery was performed. It was pathologically confirmed as a recurrent tumor (Fig. 3D).

She passed away the following month.

## 3 Discussion

Primary fallopian tube cancer is rare, constituting approximately 0.1–1.8 % of gynecological cancers [3]. In this case, based on the pathology results, a tumor was found in the thickened tubal wall, and it is presumed to be primary tubal carcinosarcoma, although a total hysterectomy had not been performed. Fallopian tubal carcinosarcomas are significantly rare, with only a few reported instances.

Preoperative diagnosis of fallopian tube cancer is challenging. There is a report indicating that only 15 % can be diagnosed before surgery [4]. Even during open surgery, there's a 30–50 % misdiagnosis rate for fallopian tubal cancer [4]. Malignancy should be considered in all cases with tubal tumors, regardless of suspicions before, during, or after surgery. Misdiagnosis can lead to significant errors in the treatment strategy. If fallopian tubal cancer is postoperatively identified, misinterpreting adhesions as inflammation may lead to omitting tumor excision, performing drainage alone instead. Particularly in cases with rapid progression, there exists the danger of a postoperative course resembling abscess formation. Thus, even when complete tumor removal is demanding, at the very least, tumor tissue biopsy is imperative.

Clinically, the triad of Latzko (watery vaginal discharge, colicky lower abdominal pain, and a pelvic mass) is well-known. However, only about 15 % meet those criteria [5]. Among imaging modalities, MRI is considered the most useful. MRI features such as a sausage-like shape, hydrosalpinx, and intrauterine fluid accumulation are characteristic to diagnose. However similar features can also be found in benign conditions. These factors contribute to the difficulty in diagnosis [3]. Therefore, during the examination of a tubal tumor, it is advisable to consider the presence of risk factors associated with fallopian tubal cancer to estimate the likelihood of malignancy. The risk factors for it include infertility and chronic inflammation [6]. This case also had a history of infertility due to tubal occlusion, which suggests the existence of chronic inflammation. It is important to inquire about the patient's history of infertility and its causes. When history taking, we should also ask about diseases that can cause chronic inflammation. Such as hydrosalpinx, pelvic inflammatory disease etc. This information influences the mindset to the surgery.

Laparoscopy was not commonly used for radical operation in advanced ovarian and fallopian tube cancers. However, recent reports have demonstrated the effectiveness of laparoscopy [7], and it is expected that laparoscopic surgery will be more actively performed for ovarian and fallopian tube cancers in the future. As a result, PSM is likely to receive increased attention in gynecological cancers. In gynecological cancers, PSM is more common in ovarian cancer [8]. The frequency is considered similarly high in fallopian tube cancer. Additionally, Carcinosarcoma is a subtype of cancer that is likely to cause PSM. This case was at high risk for PSM. To prevent PSM, procedures such as port site disinfection with iodine and meticulous washing of the specimen bag before extraction are recommended [9]. When performing laparoscopic surgery for tubal tumors, these preventive measures should be implemented. We also hope this report serves as a cautionary note regarding the potentially increasing incidence of PSM in the gynecologic field.

PSM can be directly touched by hand. A tumor that grows larger each day has a significant negative impact on the patient's mental well-being. The efficacy of tumor removal in prognosis remains uncertain. If there are other metastases or recurrences, performing PSM excision surgery does not improve prognosis. However, Excising PSM improved the psychological well-being of this patient significantly. She appeared to be relieved from a major concern. We couldn't find any papers suggesting that surgery for skin tumors improves mental health in cancer patients. However, there are studies showing that breast reduction surgery for patients with macromastia significantly improves anxiety and depression [10]. PSM patients can also have negative impact on body images. It can be said that similarly, surgery could have a positive impact on the mental health of PSM patients. If a patient expresses a desire, it might be worth considering surgery with the aim of providing psychological support. Due to the possibility of early recurrence, meticulously assessing the eligibility is essential.

## 4 Conclusion

Fallopian tube cancer is rare and challenging to diagnose. Carcinosarcoma is particularly rare and tends to progress rapidly. It is important to consider the possibility of malignancy at all times treating fallopian tubal tumors. When performing laparoscopic surgery for fallopian tubal tumors, it is advisable to consider preventive measures for PSM. PSM excision may work as a valuable psychological support for the patient.

## Author contribution

Drafting the article: Takuya Kuboya

Revising the article: Dr. Kaoru Kato

Study supervision: Dr. Junichi Sasaki

All the authors have read and agreed to the final manuscript.

## Consent

Written informed consent was obtained from the patient for publication and any accompanying images. A copy of the written consent is available for review by the Editor-in-Chief of this journal on request.

## Ethical approval

The Ethics Committee at our institution discussed the case and determined that ethical approval was not required.

## Guarantor

Dr. Junichi Sasaki.

## Sources of funding

There are no sources of funding.

## Registration of research studies

N/A.

## Declaration of competing interest

No funding was received for this research.
